# Genome Sequence Analysis of the Naphthenic Acid Degrading and Metal Resistant Bacterium *Cupriavidus gilardii* CR3

**DOI:** 10.1371/journal.pone.0132881

**Published:** 2015-08-24

**Authors:** Xiaoyu Wang, Meili Chen, Jingfa Xiao, Lirui Hao, David E. Crowley, Zhewen Zhang, Jun Yu, Ning Huang, Mingxin Huo, Jiayan Wu

**Affiliations:** 1 School of Environment Sciences, Key Laboratory of Wetland Ecology and Vegetation Restoration of National Environmental Protection, Northeast Normal University, Changchun, China; 2 The CAS Key Laboratory of Genome Sciences and Information, Beijing Institute of Genomics, Chinese Academy of Sciences, Beijing, China; 3 Department of Environmental Sciences, University of California Riverside, Riverside, California, United States of America; Belgian Nuclear Research Centre SCK•CEN, BELGIUM

## Abstract

*Cupriavidus* sp. are generally heavy metal tolerant bacteria with the ability to degrade a variety of aromatic hydrocarbon compounds, although the degradation pathways and substrate versatilities remain largely unknown. Here we studied the bacterium *Cupriavidus gilardii* strain CR3, which was isolated from a natural asphalt deposit, and which was shown to utilize naphthenic acids as a sole carbon source. Genome sequencing of *C*. *gilardii* CR3 was carried out to elucidate possible mechanisms for the naphthenic acid biodegradation. The genome of *C*. *gilardii* CR3 was composed of two circular chromosomes chr1 and chr2 of respectively 3,539,530 bp and 2,039,213 bp in size. The genome for strain CR3 encoded 4,502 putative protein-coding genes, 59 tRNA genes, and many other non-coding genes. Many genes were associated with xenobiotic biodegradation and metal resistance functions. Pathway prediction for degradation of cyclohexanecarboxylic acid, a representative naphthenic acid, suggested that naphthenic acid undergoes initial ring-cleavage, after which the ring fission products can be degraded via several plausible degradation pathways including a mechanism similar to that used for fatty acid oxidation. The final metabolic products of these pathways are unstable or volatile compounds that were not toxic to CR3. Strain CR3 was also shown to have tolerance to at least 10 heavy metals, which was mainly achieved by self-detoxification through ion efflux, metal-complexation and metal-reduction, and a powerful DNA self-repair mechanism. Our genomic analysis suggests that CR3 is well adapted to survive the harsh environment in natural asphalts containing naphthenic acids and high concentrations of heavy metals.

## Introduction

Members of the genus *Cupriavidus* are Gram-negative β-proteobacteria that are commonly isolated from environmental and human clinical sources [1]. *Cupriavidus* has a complex taxonomic history, which was addressed by Vandamme and Coenye in 2004 [2]. The genus *Cupriavidus* currently is comprised of 11 species derived from diverse ecological niches [3]. These include several heavy metal tolerant bacteria such as *C*. *metallidurans* CH34 [4], *C*. *necator* N-1 [5], *C*. *pinatubonensis* JMP134 [6], and *C*. *taiwanensis* [7]. Several genetic determinants for heavy metal resistance have been identified in *Cupriavidus* [8–9] including the *czc*, *pbr*, and *mer* clsusters, and are present in several members of the genus [8–14]. The strain with the highest number of heavy metal resistance loci is CH34 tolerating up to milli-molar concentrations of over 20 different heavy metal ions [4, 9]. Various *Cupriavidus* sp. also have the ability to degrade a range of different aromatic hydrocarbons. Strain JMP134 is a well studied pollutant-degrading bacterium [6, 14], in which pathway predictions constructed for aromatic compounds are based in multiple peripheral ring cleavage pathways and associated central reactions [15]. Three other bacteria, *C*. *eutropha* H16, *C*. necator N-1 and *C*. *taiwanensis* are reported to effectively degrade bioplastics [5, 16–17]. Another representative of this taxon, *C*. *metallidurans* strain KUA-1, can degrade the standard reference naphthenic acid, cyclohexylacetic acid (CHAA) [18].

Naphthenic acids (NAs) are complex mixtures of hydrocarbons that are predominantly comprised of saturated aliphatic and alicyclic carboxylic acids [19]. As natural components of crude petroleum, NAs range from undetectable to up to 4% concentration by weight [20]. However, NAs are thought to be the most toxic components of both refinery effluent and oil sands-processing wastewaters, and pose significant environmental and human health risks [21–22]. Microbial biodegradation has been seen as a promising solution for treatment of NA mixtures [23], and a good biodegradation efficiency has been reported for trans-4-methyl-1-cyclohexane carboxylic acid (trans-4MCHCA) [24–25]. Prior studies have shown that diverse bacteria belonging to the genera *Corynebactrium*, *Arthrobacter*, *Acinetobacter*, *Alcaligenes*, *Pseudomonas*, and *Bacillus* [26–31] utilize different commercial NAs as sole sources of carbon and energy. In contrast, only a few strains of *Cupriavidus* that are capable of degrading NAs have been identified to date. To our knowledge, there is no pathway associated with degradation of this naphthenic acid in *Cupriavidus*. Although strain KUA-1 has been reported to degrade cyclohexylacetic acid [18], the degradation pathway and its substrate versatility remain largely unknown.

Here, we report the complete genome sequence and analysis of a novel *C*. *gilardii s*train CR3, which provides the foundation for elucidating the mechanisms for naphthenic acid biodegradation and heavy metal resistance. Trans-4MCHCA was chosen to elaborate the bacterial degradation pathways. Genomic analysis of *C*. *gilardii* CR3 indicated that this bacterium degraded the cyclohexanecarboxylic acid via a ring-cleavage pathway [23, 30]. The acyclic-open chain compound was then further oxidized by β-oxidation, which involves a fatty acid β-oxidation module. We propose a probable degradation pathway and corresponding versatility. Besides its ability to degrade naphthenic acid, metal resistance was also identified in CR3. This strain is tolerant to no less than ten heavy metals, which was mainly achieved by high-performance ion efflux, metal-complexation and metal-reduction, and a powerful self-repair mechanism.

## Materials and Methods

### Isolation of strain CR3

*C*. *gilardii* CR3 was isolated from the Rancho La Brea Tar Pits 91, Los Angeles, CA, USA. The geographical coordinates is Lat 34° 3' 46.1268'' N, Lon -118° 21' 21.1716'' W. Kim and Crowley completed a comprehensive survey of this same location earlier, which included a characterization of its chemical properties and microbial diversity [32]. Samples of asphalt-bearing soil were obtained by scraping away the upper surface of the asphalt with a sterile knife and were placed into sterile plastic tubes and transported to the laboratory where they were stored at 4°C until processing. For isolation of NA-biodegrading bacteria, 95 mL of sterilized Milli-Q water were added to 5 g of asphalt-bearing soil in a 250 mL Erlenmeyer flask and shaken for 24 h. The resulting cell suspension culture was spread onto MSM agar plates amended with trans-4-methyl-1-cyclohexane carboxylic acid (final concentrations of 150 mg L^-1^), and incubated for 5 days at 28°C. One strain, designated as strain CR3, was selected on the basis of its rapid growth on trans-4-methyl-1-cyclohexane carboxylic acid. Individual colonies were selected randomly and purified by single colony isolation after triple re-streaking on LB agar medium. The strain also grows on tryptic soy medium.

### Isolation identification and extraction of genomic DNA

Identification of strain CR3 was performed based on morphological, physiological, and phylogenetic characteristics. The morphological and physiological characteristics of the strain were performed based on Bergey’s manual of systematic bacteriology (second edition, 2004). To determine the 16S rRNA gene sequences and to establish DNA libraries for genome sequencing, the genomic DNA of strain CR3 was extracted using a DNA extraction kit (TakaRa DNAiso Reagent code: D305A (TaKaRa Biotechnology Company Limited, Dalian, China)). To confirm the identities of the isolates, PCR amplification and sequencing of the 16S rRNA gene were performed. The 16S rRNA genes were PCR-amplified from the genomic DNA using the bacterial universal primer set 27f (5'-AGA GTT TGA TCC TGG CTC AG-3') and 1492r (5'-GGC TAC CTT GTT ACG ACT T-3'). The 16S rRNA gene sequence of strain CR3 was compared with those in EzTaxon-e database (http://www.ezbiocloud.net/eztaxon) [33] and GenBank database (http://www.ncbi.nlm.nih.gov/blast) using BLAST [34] to identify closely related bacteria.

### Genome sequencing and genome assembly

In this study, CR3 cells were sequenced by Illumina HiSeq2000 (Illumina, San Francisco, USA) and Pacific Biosciences II (Pacific Biosciences, San Francisco, USA) sequencing technologies. Three DNA libraries for Illumina sequencing were constructed, including a 180 bp pair-end library, and 1–2 kb and 3–5 kb mate-pair libraries. We generated 50,065,980 pair-end reads (2×101 bp), and 128,546,539 mate-pair reads (2×101 bp). High quality of sequencing reads is a prerequisite for good genome assembly. To this end, we carried out a filtering process to filter low quality reads (reads with excess “N” and low quality score), duplication reads, and adaptor contamination reads. After filtering, three libraries yielded approximately 3126-fold coverage by high quality reads. High quality reads were assembled by SOAPdenovo v2.04 [35]. Inner gaps that emerged in the scaffolding were filled with GapCloser [36]. High quality HiSeq reads were assembled into 21 contigs and 4 scaffolds. We used Pacific Biosciences SMART analysis software 1.2 to generate long ‘filtered sub-reads’ from the instrument, which are high quality reads. Filtered sub-reads were generated through the following primary analysis: removal of adaptors, removal of low quality bases, and removal of short reads. Here, we call sub-reads as ‘reads’, and never refer to raw reads. After filtering, SMRT cell sequencing generated 52,939 long reads, and the sum of bases was 296,078,365 bases. Average read length was up to 5,592 bp. SMRT cells yielded approximately 44-fold coverage. All PacBio reads were assembled into 2 polished contigs using HGAP v3 [37]. One of them was able to be direct circularization. The other one contained a circularization gap. Circularization gap was filled with PBJelly [38]. The filled gap sequence errors were corrected using HiSeq assembly results. This Whole Genome Shotgun project has been deposited at DDBJ/EMBL/GenBank under the accession CP010516-CP010517.

### Genome annotation and analysis

Open reading frames (ORFs) were predicted using GeneMarks [39] and Glimmer 3.0 [40]. The predicted ORFs were validated by BLAST [34], which was used to search for homologous genes. Protein domains were scanned by InterProScan [41]. Functional genes that were investigated as having possible roles in metabolic pathways were reconstructed by KAAS (a module of KEGG) [42]. Phylogenetic classification of proteins that are encoded in the CR3 genome were based on functions of clusters of orthologus groups (COG) [43]. Transfer RNA (tRNA) genes in the genomic sequence were detected by tRNAscan-SE [44]. Ribosomal RNAs were searched using RNAmmer [45].

Seven published genome sequences of *Cupriavidus* strains were collected (published before 2013) to analyze species phylogeny and to generate a synteny plot. These strains included *C*. *pinatubonensis* JMP134 [6], *C*. *metallidurans* strain CH34 [4], *C*. *necator* N-1 [5], *C*. *taiwanensis* [7], *C*. *eutropha* H16 [17], *R*. *eutropha* HPC(L) and *C*. *basilensis* OR16 [11]. We also gathered other public and completed 16S rRNA genes from NCBI to present a full taxonomic analysis. A rooted phylogenetic tree based on 16S rRNA gene sequence similarity of the genera *Cupriavidus* was performed using Mega 6.0 [46]. Cluster analysis was based on the neighbour-joining method with the closely related bacterium *Ralstonia pickettii* 12D [2] as the outgroup root. Synteny plots were analyzed by Mauve v2.3.1 [47]. Rooted phylogenetic tree based on gene gain and loss was performed by PGAP [48]. Phylogenetic trees were visualized by TreeView [49]. Ortholog gene clusters were identified by PGAP [48]. Core genes were identified as genes shared by all strains. Unique genes were identified as genes that were unique to one strain. Likewise, replicon chr1 core genes were identified as genes shared by all strains and limited in replicon chr1. Replicon chr1 unique genes were identified as genes that were unique to one strain and limited to replicon chr1. The same criteria were applied to replicon chr2 core genes and replicon chr2 unique genes. Functional distribution over chr1 and chr2 was reflected by the relative ratio of COG classes, which represented the normalized ratio of percentages of CDS numbers per replicon in a COG class [4]. Unique genes and genome genes in each GO category were counted. A statistical p value of unique genes enriched in each GO category was calculated by the chi-square test. GO functions having fewer than 3 genes were removed and GO functions in which the percent of unique genes was 1% or more less than the percent of the genome genes were also removed. The unique-gene percent was defined as the percent of unique genes in a GO function divided by the total of all unique genes. The genome gene percent was defined as the percent of genome genes in a GO function divided by the total of all genome genes. GO enrichment was considered significant when p values were <0.05. In the KEGG pathway enrichment analysis, pathways were removed if the gene count in CR3 was less than 5 or if the gene count discrepancy between CR3 and the *maximum* gene count of other 7 *Cupriavidus* strains was less than 5. Unique-gene pathway enrichment was selected as above by GO function enrichment analysis. COG enrichment analysis of unique genes was also performed as above by GO function enrichment analysis. Possible enzyme-catalyzed metabolic pathways for multi-step reactions involved in naphthenic acid degradation were predicted by PathPred [50], which was based on the local RDM pattern (KEGG atom type changes at the reaction center (R), the difference region (D), and the matched region (M) for each reactant pair.) match and the global chemical structure alignment. To analyze enzyme-encoding gene organization, we counted enzyme-encoding gene percent that genes located at gene clusters. Genes that genomic locations were adjacent formed a gene cluster.

### Determination of minimum inhibitory concentration (MIC)

MIC values for nine heavy metals (Ag, Cd, Co, Cr, Cu, Hg, Ni, Pb and Zn) against *C*. *gilardii* CR3 were determined using broth cultures. The 1 M stock solutions of heavy metal salts were made from using analytical grade chemicals and were sterilized by filtration. MIC was firstly determined using a two-fold dilution method, which generated an appropriate MIC range. Afterwards serial continuous concentration dilution experiments were carried out to identify specific MIC values based on the pre-defined MIC range. The media used for MIC was Tris-buffered mineral salts medium containing 0.2% (m/m) sodium gluconate adopted by Mergeay et al. [51]. The optical density of cultures was measured at 600nm (OD_600_) using a UV-visible spectrometer (T6, Beijing Purkinje General Instrument Co. Ltd., Beijing, China). Pre-cultured *C*. *gilardii* CR3 (OD_600_ = 1) was inoculated to a tube with media, cultivated with shaking at 160rpm (30°C) for 12h. Heavy metal solutions (4% weight/volume) were added to the corresponding tubes, and the cells then further cultivated for 96h. Cultures containing media inoculated with *C*. *gilardii* CR3 but without heavy metals served as the controls. The OD_600_ of the cultures were measured from 0h to 96h at 12 h intervals.

## Results and Discussion

*Cupriavidus* strains are well known for their ability to inhabit environments containing toxic heavy metals, especially those containing Cu^2+^, which lead to their genus name (i.e. “copper-loving”). Members of this genus also are able to degrade many different complex compounds [5–6, 15–18], such as polyhydroxybutyrate, which is degraded by strain H16 [17], chloroaromatics degraded by strain JMP134, mainly based on the β-ketoadipate pathway [15], and CHAA which can be degraded by strain KUA-1 [18]. However, the degradation pathways for these substances are unclear and their normal substrates are largely unknown. *C*. *gilardii* strain CR3, studied here, was isolated from a natural asphalt seep located at the Rancho La Brea Tar Pits (Los Angeles, CA), and was shown to use 4MCHCA and other similar molecules with branched alkyl groups (-(CH_2_)_m_CH_3_) as sole sources of carbon and energy for growth. This has never been shown for other strains of *Cupriavidus*. This establishes strain CR3 as a potential microbial agent to biodegrade either model NAs or commercially available NA compounds, which are among the most toxic components in both refinery effluent and oil sands process wastewaters, and also establishes this strain as a petroleum degrading bacterium with heavy metal resistance.

### Isolation and description of *Cupriavidus gilardii* CR3

Four microorganisms capable of using trans-4MCHCA as a sole carbon and energy for growth were isolated from the Lar Brea Tar Pits (Los Aageles, CA). From these, we finally selected strain CR3 for determination of its naphthenic acid biodegradation pathway based on its high growth rate on this carboxylic acid. Cells of strain CR3 were observed to be aerobic, rod-shaped, and Gram-negative. The colonies produced by this bacterium (3–5 mm diameter) were smooth, circular convex, wet, and light yellowish in color for cells grown on LB agar medium for 2 days at 28°C. The strain was positive for catalase and oxidase.

The 16S rRNA gene sequence (1520 bp) of strain CR3 (NCBI GeneBank accession JX945578) was obtained. The BLAST search results based on EzTaxon and GenBank databases both indicated that strain CR3 belonged to *Cupriavidus*. The 16S rRNA gene sequence of strain CR3 was 100% identical to *C*. *gilardii* CIP 105966. Morphological, physiological and phylogenetic properties all suggested that strain CR3 was *C*. *gilardii*.

### Genome structure and general features

**Fig 1:**
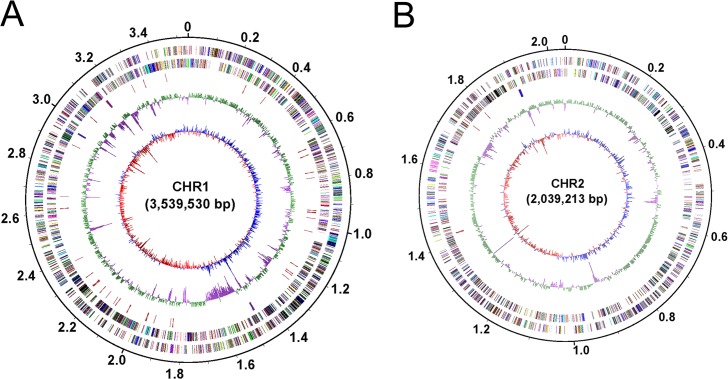
Circular representation of the two replicons of *C*. *gilardii* CR3. **(A). Chromosome 1; (B). Chromosome 2.** Circles display from the inside outwards, (1) circle 1: GC-skew (G-C/G+C ratio) using a 3 kb window; (2) circle 2: GC-content using a 3 kb window; (3) circle 3: RNA genes; (4) circle 4: COG assignments for predicted CDSs on the minus strand; (5) circle 5: COG assignments for predicted CDSs on the plus strand; (6) circle 6: scale in Mb.

The complete genome of CR3 contained 2 circular chromosomes without any inner gaps. The size of chromosome 1 (chr1) was 3,539,530 bp and chromosome 2 (chr2) was 2,039,213 bp, and summed to 5,578,743 bp. The GC content of CR3 genome was 67.55%. A total of 4,502 putative coding sequences (CDSs) were validated by homology search. The complete genome contained 59 tRNA genes and 4 *rrf*-*rrl*-trnI-trnA-*rrs* operons (with rrf, rrl, and rrs coding for respective 5S, 23S, and 16S rRNA). There were 3,862 CDSs that were assigned to one or more function classes (Fig 1, Table 1). The GC content of two replicons was very similar (Fig 1). Approximately 86% of 4,501 CDSs could be assigned a putative function based on function annotation. The general features of two replicons were displayed in Table 1.

**Table 1:** General features of the *C*. *gilardii* CR3 genome.

	Chromosome 1	Chromosome 2	Genome
**Size (bp)**	3,539,530	2,039,213	5,578,743
**GC content**	67.40%	67.81%	67.55%
**tRNA**	52	7	59
**rRNA operons**	3	1	4
**Total number of CDSs**	2,874	1,627	4,501
**Overlapping CDSs**	545	342	887
**CDSs with assigned function**	2,504	1,358	3,862
**Hypothetical proteins**	370	269	639
**CDSs assigned to COGs**	2,130	1,104	3,234
**CDSs assigned to KEGG Ontology**	1,687	698	2,385
**CDSs assigned to GO function**	1,740	923	2,663

**Fig 2:**
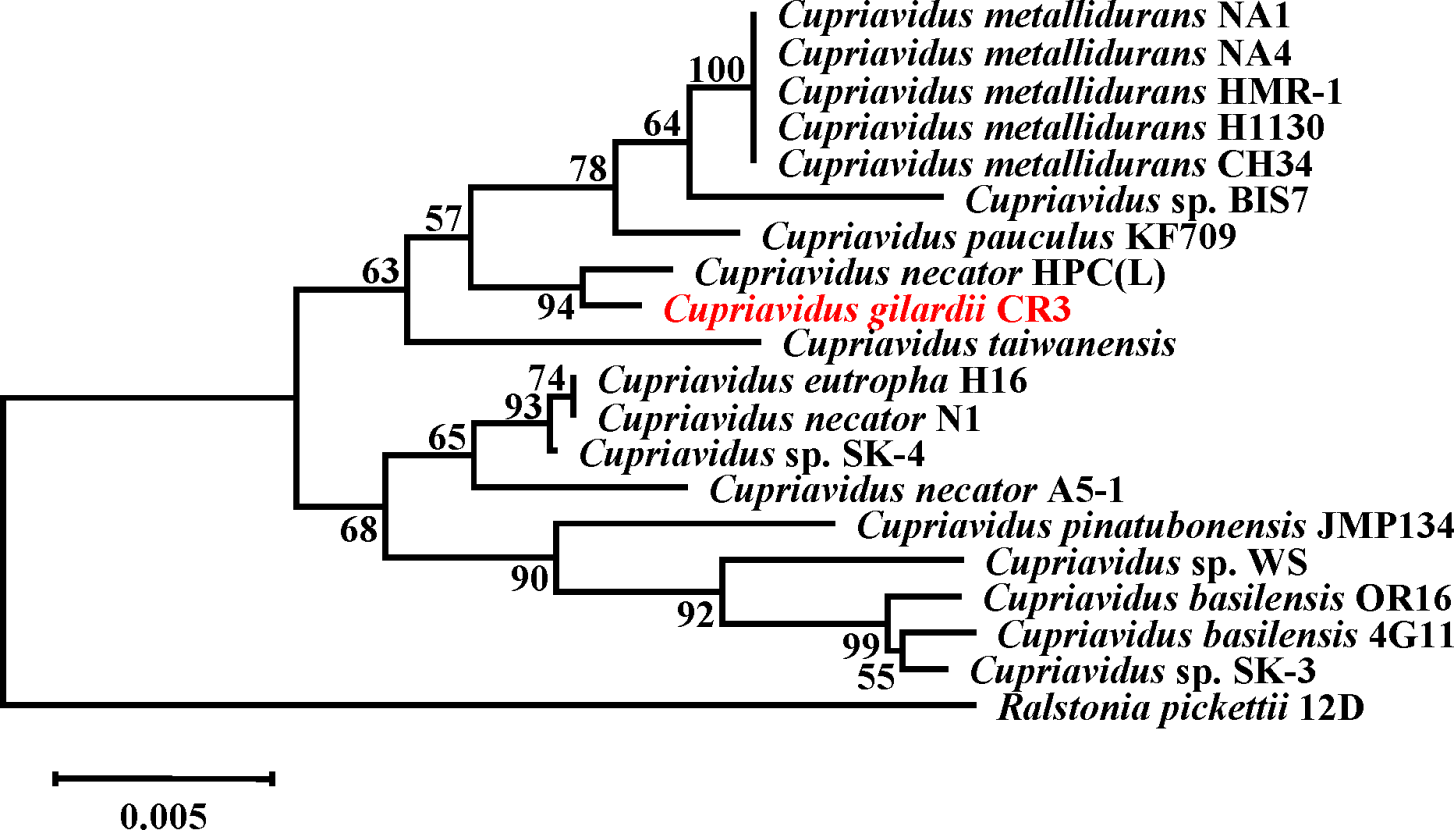
Rooted phylogenetic tree based on 16S rRNA gene sequence similarity of the genus *Cupriavidus*. Cluster analysis was based upon the neighbour-joining method with *Ralstonia pickettii* 12D as the outlier. The scale bar represents 5‰ sequence divergence. Numbers at branch-points are percentages of 1000 bootstrap resamplings that support the tree topology.

Phylogenetic analyses based on 16S rRNA gene sequences suggested *C*. *gilardii* CR3 fit firmly into the genus *Cupriavidus* strain. The result indicated that its closest relative was *C*. *necator* HPC (L) (Fig 2). The relationship between the CR3 and HPC (L) strains was also shown in their genome composition analysis. As shown in Table 2, other *Cupriavidus* genome sizes [4–7, 11, 17] range from 5.49 Mb to 8.55 Mb, and the smallest genome is HPC (L); CR3 comes second. Whereas, GC content of all these other members of *Cupriavidus* varies from 63.53% to 67.54%, and the highest GC content genome is HPC (L). And strain CR3 has the GC content at 67.55%. Gene number in other genomes ranges from 4,990 to 7,915, and the fewest gene number genomes is HPC (L). And strain CR3 contained only 4,572 genes. Both results of GC content and gene number indicate the close relationship between CR3 and HPC (L). Moreover, the CR3 genome was composed of only two chromosomes, while other finished genomes are composed of two chromosomes and at least one plasmid.

**Table 2:** Genome structure comparison between CR3 and seven other published *Cupriavidus* bacteria.

Bacterium	Status	Genome Size (M)	GC content (%)	Chr	Plasmid	Protein	rRNA	tRNA	Total	Reference or download source
***Cupriavidus eutropha* H16**	Finished	7.42	66.34	2	1	6,626	15	58	6,699	[17]
***Cupriavidus taiwanensis***	Finished	6.48	66.99	2	1	5,896	15	63	5,974	[7]
***Cupriavidus necator* N-1**	Finished	8.48	65.47	2	2	7,832	15	68	7,915	[5]
***Cupriavidus metallidurans* CH34**	Finished	6.91	63.53	2	2	6,477	12	62	6,551	[4]
***Cupriavidus pinatubonensis* JMP134**	Finished	7.26	64.43	2	2	6,446	18	66	6,530	[6]
***Cupriavidus basilensis* OR16**	Draft	8.55	65.39	-	-	7,534	5	61	7,600	[11]
***Cupriavidus necator* HPC(L)**	Draft	5.49	67.54	-	-	4,931	6	53	4,990	ftp://ftp.ncbi.nlm.nih.gov/genomes/Bacteria_DRAFT/Cupriavidus_necator_HPC_L__uid180939/
***Cupriavidus gilardii* CR3**	Finished	5.58	67.55	2	0	4,501	12	59	4,572	This study

**Fig 3:**
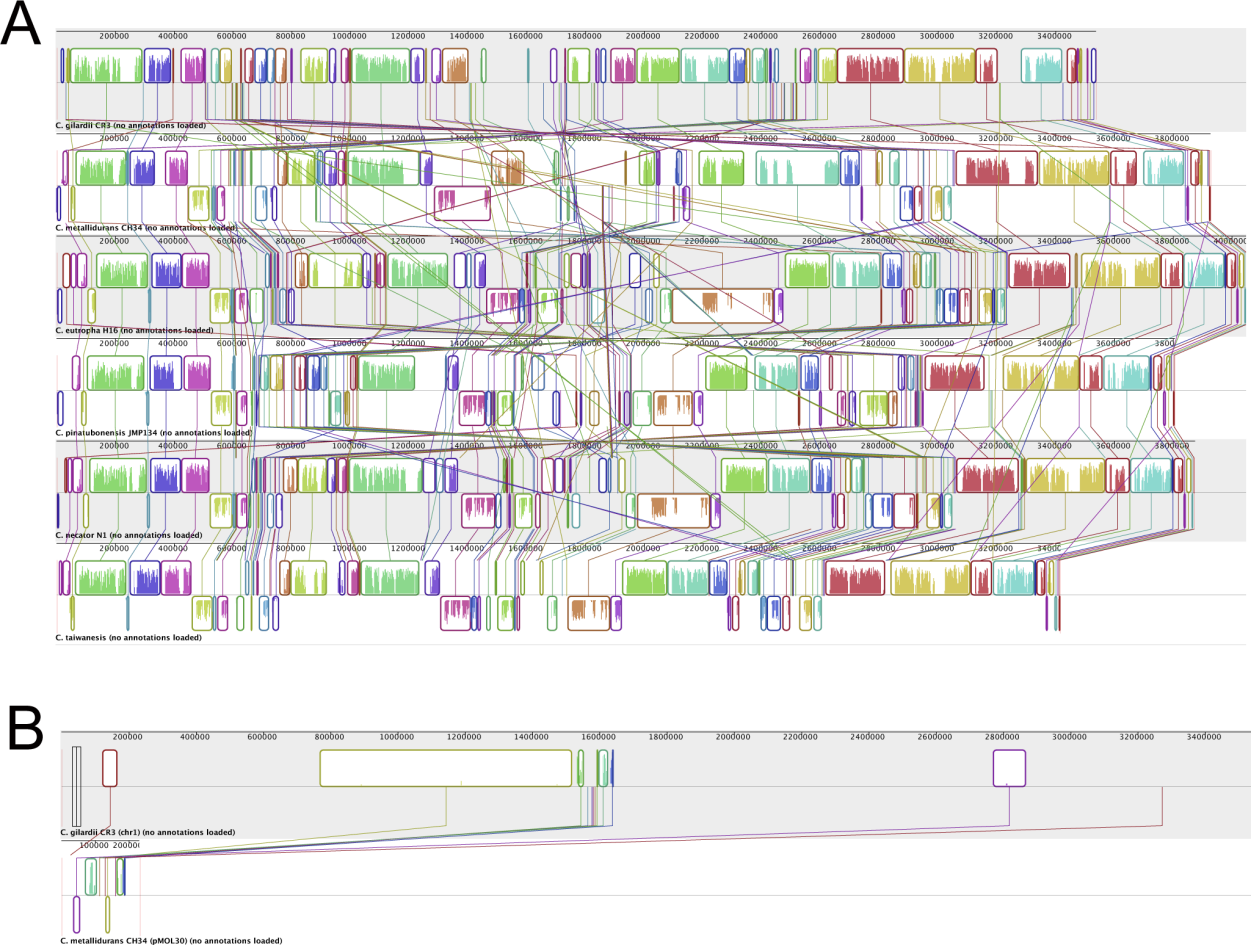
Synteny plot analysis of *Cupriavidus* replicons. (A) Synteny plot analysis of Chr1 from *C*. *galardii* CR3 and from 5 other finished *Cupriavidus* strains; (B) Synteny plot analysis of Chr 1 from *C*. *galardii* CR3 and pMOL30 from *C*. *metallidurans* CH34.

To explore the genome structure of strain CR3, we carried out synteny analysis on CR3 and all other reported *Cupriavidus* genomes. CR3 genome sequences clearly showed synteny in chr1 with other finished strains (Fig 3A) but lack of synteny in chr2 (S1 Fig). The synteny phenomenon is similar to JMP134 [6]. The sequence in the region chr1:1,518,182–1,620,123 of CR3 showed some successive synteny with a plasmid of CH34 (pMOL30) (Fig 3B), which has not been previously reported in other *Cupriavidus* strains. The synteny analysis reveals conservation of chr1 sequence composition since it's an ancestral replicon [4, 6]. Plasmid fragment recombination into chr1 was generally incompact, whereas the synteny region between CR3 chr1 and CH34 plasmid pMOL30 tended to be relatively concentrated. Moreover, CH34 was the closest relative to CR3 among the five finished *Cupriavidus* strains to which it was compared. The lack of synteny in the smaller replicon chr2 probably originated from ancestral plasmids during the evolution of *Cupriavidus* and appeared to have evolved with frequent recombination, gene duplication and large numbers of transfered genes [4, 6]. The chr2 replicon displayed conserved repA-parA-parB (CR3_2936, 2938, 2939) gene structure, implying that it may have derived from plasmid-like partitioning [4–7, 17]. This indicates that the plasmid fragment recombination mechanism in replicon chr1 and replicon chr2 is quite different, not only in recombination frequency, but also in the source of plasmid fragments.

**Fig 4:**
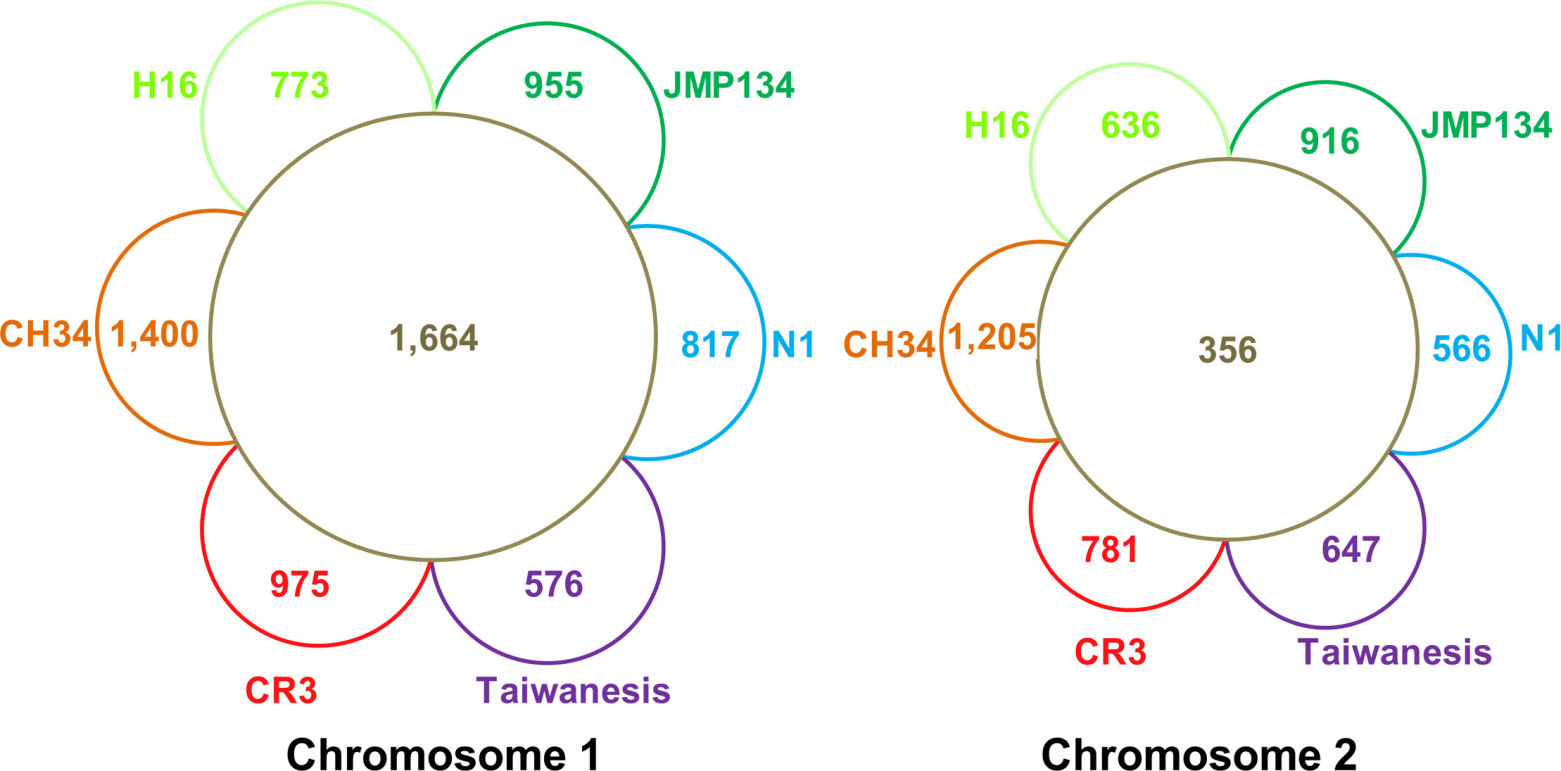
Comparative analysis of gene distribution in two chromosomes. Inner ring shows the number of ortholog gene clusters that are shared by all species based on PGAP result. Outer semi-ring shows the number of ortholog gene clusters that are unique to one strain based on PGAP result. Abbreviation: CR3, *C*. *gilardii* CR3; CH34, *C*. *metallidurans* CH34; H16, *C*. *eutropha* H16; JMP134, *C*. *pinatubonensis* JMP134; N1, *C*. *necator* N1; Taiwanesis, *C*. *taiwanesis*.

**Fig 5:**
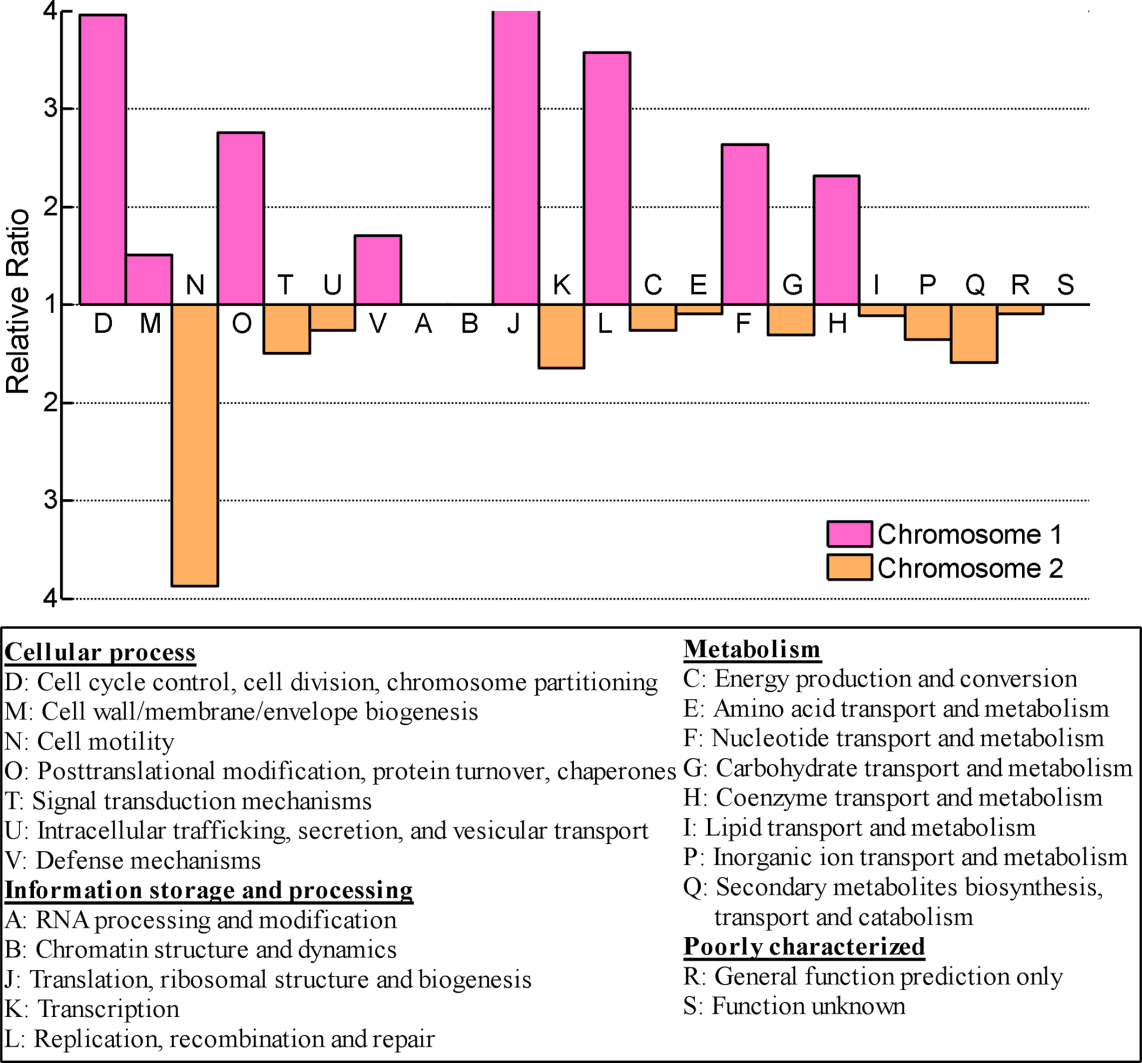
Functional distribution over two replicons based on COG classification. The scale represents the relative ratio of CDS numbers per gene source (i.e. the ratio of percentages for each class per replicon).

Gene distribution analyses also indicated that CR3 chr1 constitutes the ancestral replicon. Compared with five other finished *Cupriavidus* strains, the core gene number in chr1, 1,672, was almost 5X larger than chr2, 356 (Fig 4). For each replicon in CR3, replicon core genes accounted for 58.91% chr1 genes and 22.28% chr2 genes respectively. Replicon unique genes accounted for 32.66% chr1 genes and 46.81% chr2 genes. In addition, chr2 replicon contained several genes paralogous to chr1 genes, such as membrane protein *ompC* (CR3_4033), cobalt-zinc-cadmium resistance protein *czcB* (CR3_3629), ABC transporter *tauB* (CR3_3580), aromatic amino acid aminotransferase *tyrB* (CR3_3642), and others. The core genes were generally associated with housekeeping gene functions, while the unique genes were associated with special features. The diversity in core gene number and unique gene number distribution in the two replicons also reflected the diversity of gene function distribution. In strain CR3, the main replicon chr1 carried most of the essential housekeeping gene functions, including those for translation, ribosome production, DNA replication, DNA repair, protein processing, cell cycle, cell division and cell component etc. (Fig 5). In contrast, the smaller replicon chr2 carried functions mainly related to adaptation and survival, including signal transduction, energy metabolism and ion transduction. The bias of replicon function distribution in CR3 is more obvious than in CH34. CR3 chr2 consists of more genes relevant to energy metabolism than chr1 (classes C, E, G), while CH34 does not [4].

Compared with seven other *Cupriavidus* strains, 897 genes were unique in CR3. To explore the specific character of CR3, we carried out GO function enrichment analysis (S2 Fig), KEGG pathway analysis (S3 Fig), and COG function classification enrichment analysis (S4 Fig) of whole genome genes and unique genes in CR3. Function annotation analysis revealed many of these unique genes in CR3 were enriched in genes relevant to biodegradation of complex compounds, signal interaction with its surroundings, and environmental adaption, which were speculated to be associated with its NAs biodegradation potential and heavy metal resistance.

### Naphthenic acid degradation

**Fig 6:**
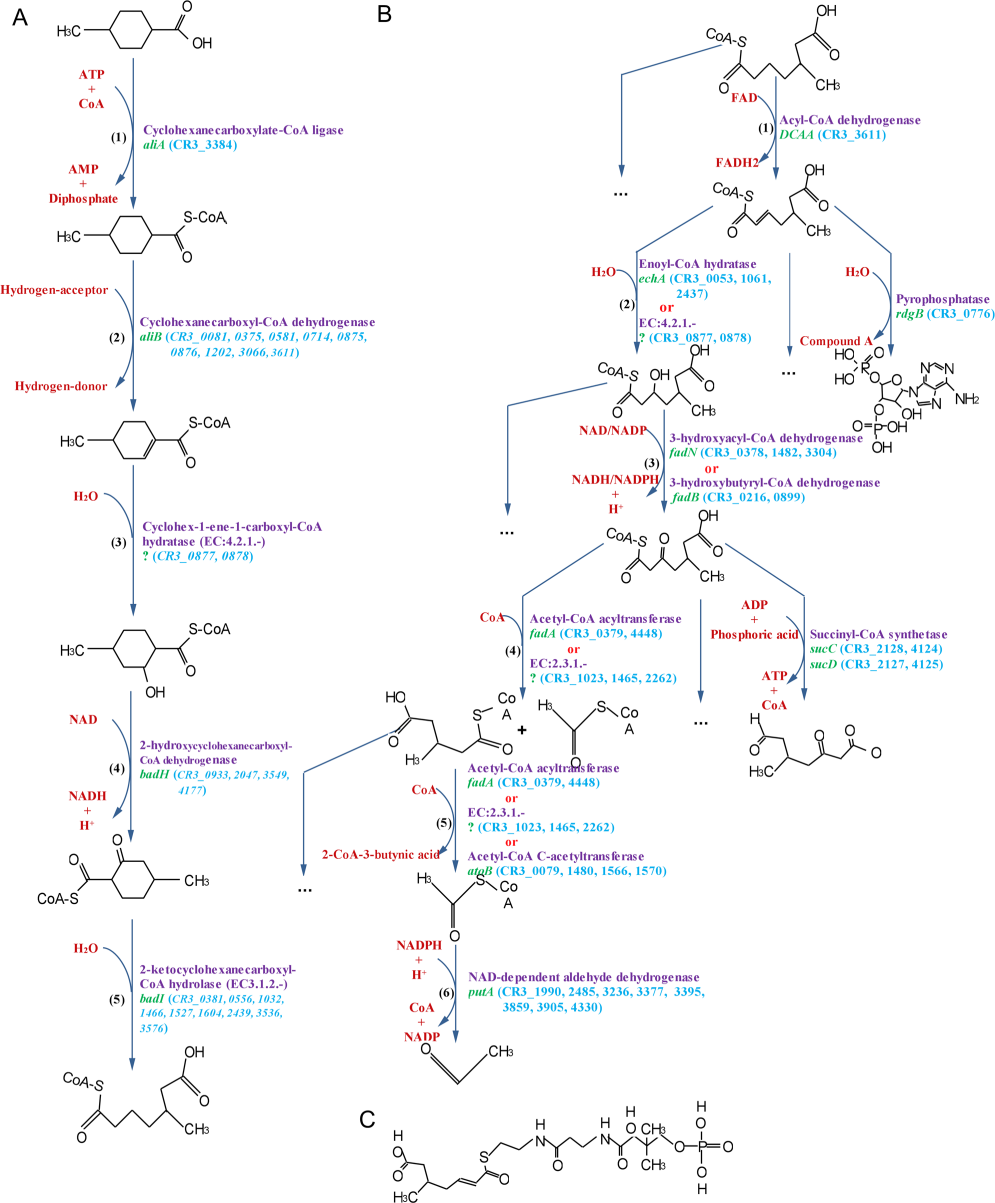
Predicted naphthenic acid degradation pathway in CR3. (A) Peripheral ring-cleavage pathway; (B) Central oxidative degradation pathways; (C) The structure of compound A. Compounds in red are substrates. Enzyme genes are marked on the right of arrows, including gene description (purple color), *gene symbol* (green color) and gene identifications (light blue color). If CR3 genome only encoded isoenzyme gene, gene identifications were marked in *italic*. Isoenzyme gene were defined as a gene assigned to a same enzyme class with the same EC NO. And enzyme genes with unknown gene symbol were marked as ‘?’.

Strain CR3 could use 4MCHCA and other compounds having different numbers of branched alkyl groups (-(CH_2_)_m_CH_3_) as sole carbon and energy sources for growth. 4MCHCA is a monocyclic saturated carboxylic acid and a general representative of nonaromatic naphthenic acids (NAs). NAs biodegradation by CR3 was observed to be an aerobic process. The metabolic pathways for 4MCHCA degradation in strain CR3 were predicted by PathPred [50], which uses a reactant pair library to obtain plausible pathways of multi-step reactions. There were 17 matched aerobic degradation pathways, which contained two main processes: the peripheral ring-cleavage step and central oxidative degradation. The peripheral ring-cleavage process in CR3 was similar to the proposed process for aerobic oxidation of cyclohexane carboxylic acid by aerobic oxidation pathways in *Pseudominas putida* [30] (Fig 6A). The central pathways functioned for transformation of acyclic-open chain compounds into a few volatile or unstable compounds, yielding products such as acetaldehyde and adenosine compounds, which were subsequently oxidized via core metabolic pathways. The degradation mechanism was consistent for NAs with different branched alkyl groups in CR3.

The peripheral ring-cleavage pathway was proposed to include five main reactions (Fig 6A, Table 3) as follows: 1) Acylation: acyl-CoA connected to 4MCHCA, and substituted carboxylic acid in the catalysis of cyclohexanecarboxylate-CoA ligase *aliA* (CR3_3384). 2) Vinylation: the saturated bond at the α position can be destroyed under the action of the cyclohexanecarboxyl-CoA dehydrogenase *aliB* (isoenzyme genes CR3_0081, 0375, 0581, 0714, 0875, 0876, 1202, 3066, 3611), forming an unsaturated ethylenic bond with double bonds. 3) Alcoholization: 4-methyl-cyclohex-1-ene-carboxyl-CoA can be transformed to 4-methyl-2-hydroxycyclohexane-carboxyl-CoA by the enzyme cyclohex-1-ene-carboxyl-CoA hydratase (EC: 4.2.1.-, isoenzyme genes CR3_0877, 0878), which destroys the ethylenic bond and attaches a hydroxyl group to the molecule. The production of a hydroxylated intermediate is a general metabolic process that is commonly involved in aerobic degradation of NAs. 4) Aldehyde: 4-methyl-2-hydroxycyclohexane-carboxyl-CoA can be transformed to yield 4-methyl-2-ketocyclohexane-carboxyl-CoA under the action of the 2-hydroxycyclohexane-carboxyl-CoA dehydrogenase *badH* (isoenzyme genes CR3_0933, 2047, 3549, 4177), which oxidized the hydroxyl group into an aldehyde. 5) Ortho-ring cleavage: 4-methyl-2-ketocyclohexane-carboxyl-CoA can be transformed to the compound 5-methyl-6-carboxy-hexanoyl-CoA under the action of the enzyme 2-ketocyclohexanecarboxyl-CoA hydrolase *badI* (isoenzyme genes CR3_0381, 0556, 1032, 1466, 1527, 1604, 2439, 3536, 3576), then the saturated ring can be converted into an acyclic-open chain. The participation of a variety of enzymes through a series of reactions would cleave the saturated NA ring to an acyclic-open chain. Subsequently, the acyclic-open chain can be further oxidized into a few volatile and unstable compounds.

**Table 3:** NAs degradation genes.

Process	Step	Gene symbol	EC No.	Definition	Gene(s) identification NO.	Isoenzyme gene(s) identification NO.^1^
**Ring-cleavage**	Acylation	*aliA*	EC:6.2.1.-	Cyclohexanecarboxylate-CoA ligase	CR3_3384	CR3_0434, 0592, 1107, 1488, 2610, 3988
	Vinylation	*aliB*	EC:1.3.99.-	Cyclohexanecarboxyl-CoA dehydrogenase	/	CR3_0081, 0375, 0581, 0714, 0875, 0876, 1202, 3066, 3611
	Alcoholization	-^2^	EC:4.2.1.-	Cyclohex-1-ene-1-carboxyl-CoA hydratase	/	CR3_0877, 0878
	Aldehyde	*badH*	EC:1.1.1.-	2-hydroxycyclohexanecarboxyl-CoA dehydrogenase	/	CR3_0933, 2047, 3549, 4177
	Ortho-ring cleavage	*badI*	EC:3.1.2.-	2-ketocyclohexanecarboxyl-CoA hydrolase	/	CR3_0381, 0556, 1032, 1466, 1527, 1604, 2439, 3536, 3576
**Acyclic-open chain degradation**	Vinylation	*DCAA*	EC:1.3.99.-	Acyl-CoA dehydrogenase	CR3_3611	CR3_0081, 0375, 0581, 0714, 0875, 0876, 1202, 3066
	Alcoholization	*echA*	EC:4.2.1.17	Enoyl-CoA hydratase	CR3_0053, 1061, 2437	/
		-^2^	EC:4.2.1.-	-	CR3_0877 CR3_0878	/
	Aldehyde	*fadN* ^3^	EC:1.1.1.35	3-hydroxyacyl-CoA dehydrogenase	CR3_0378, 1482, 3304	/
		*fadB*	EC:1.1.1.157	3-hydroxybutyryl-CoA dehydrogenase	CR3_0216, 0899	/
	Deacetylation	*fadA*	EC:2.3.1.16	Acetyl-CoA acyltransferase	CR3_0379, 4448	/
		-^2^	EC:2.3.1.-	-	CR3_1023, 1465, 2262	CR3_0137, 0138, 0139, 1571, 2147, 2925, 2964, 4321
	Further deacetylation	*fadA*	EC:2.3.1.16	Acetyl-CoA acyltransferase	CR3_0379, 4448	/
		-^2^	EC:2.3.1.-	-	CR3_1023, 1465, 2262	CR3_0137, 0138, 0139, 1571, 2147, 2925, 2964, 4321
		*atoB*	EC:2.3.1.9	Acetyl-CoA C-acetyltransferase	CR3_0079, 1480, 1566, 1570	/
	Deacylation	*putA* ^4^	EC:1.2.1.3^3^	NAD-dependent aldehyde dehydrogenase^4^	CR3_1990, 2485, 3236, 3377, 3395, 3859, 3905, 4330^4^	/

1. Isoenzyme genes were defined as a gene assigned to a same enzyme class with the same EC NO.

2. Gene symbol was unknown.

3. The *fadN* gene was assigned to a same enzyme class and definition as HADH gene, except KEGG ko orthology.

4. The CR3 genome did not encode an acetaldehyde dehydrogenase (EC1.2.1.10), but encoded week specific enzyme of aldehyde dehydrogenase (EC1.2.1.3).

We identified several possible central pathways to transform 5-methyl-6-carboxy-hexanoyl-CoA into a few volatile and unstable compounds. For example, the end-product of acetaldehyde metabolism in CR3 was predicted to pass through following reactions (Fig 6B and 6C, Table 3): 1) Vinylation: 2-position saturated bond of 5-methyl-6-carboxy-hexanoyl-CoA can be destroyed under the action of the acyl-CoA dehydrogenase *DCAA* (CR3_3611), forming an unsaturated ethylenic bond with double bonds. 2) Alcoholization: involving either enoyl-CoA hydratase *echA* (CR3_0053, 1061, 2437) or a type of hydro-lyases enzyme (EC:4.2.1.-, CR3_0877 CR3_0878), the double ethylenic bond in 2-ene-5-methyl-6-carboxy-hexanoyl-CoA can be destroyed, while an hydroxyl was connected. 3) Aldehyde: 2-position hydroxyl can be oxidized into aldehyde, which may be mediated by 3-hydroxyacyl-CoA dehydrogenase *fadN* (isoenzyme gene of HADH gene, CR3_0378, 1482, 3304) or 3-hydroxybutyryl-CoA dehydrogenase *fadB* (CR3_0216, 0899). 3-hydroxyl-5-methyl-6-carboxy-hexanoyl-CoA can be oxidized into 3-aldehyde-5-methyl-6-carboxy-hexanoyl-CoA. 4) Deacetylation: acetyl-CoA can be removed from the long chain compound 3-aldehyde-5-methyl-6-carboxy-hexanoyl-CoA under a catalytic reaction mediated by acetyl-CoA acyltransferase *fadA* (CR3_0379, 4448) or a type of acyltransferases enzyme (EC:2.3.1.-, CR3_1023, 1465, 2262) to generate 3-methyl-4-carboxy-hexanoyl-CoA and acetyl-CoA. Acetyl-CoA could then be degraded in step 6). 5) Further deacetylation: acetyl-CoA can be further broken from 3-methyl-4-carboxy-hexanoyl-CoA via the enzyme acetyl-CoA acyltransferase *fadA* (CR3_0379, 4448), or a type of acyltransferases enzyme (EC:2.3.1.-, CR3_1023, 1465, 2262), or acetyl-CoA C-acetyltransferase *atoB* (CR3_0079, 1480, 1566, 1570). 6) Deacylation: acetyl-CoA could be degraded into acetaldehyde by the enzyme NAD-dependent aldehyde dehydrogenase *putA* (CR3_1990, 2485, 3236, 3377, 3395, 3859, 3905, 4330), which is a highly volatile compound. The end product was not toxic to CR3. The acyclic-open chain degradation pathway is similar to β-oxidation module in fatty acid metabolism. Multiple plausible pathways and multiple genes encoding this suite of enzymes permit CR3 to degrade NAs for use as a carbon and energy source for growth.

Likewise, there were 18 plausible reaction pathways to degrade 4-pentyl-cyclohexane carboxylic acid. The distinction between the two broad groups of metabolic pathways mainly involved different enzymes that are used to catalyze branched alkyl groups levels for different NAs. The central pathway degraded nonaromatic NAs consisted of two processes: ring cleavage and further oxidation to volatile compounds. These two processes both included four similar steps, which were dehydrogenation to form an ethylenic bond, a hydratase to produce alcohol, oxidation of the alcohol to an aldehyde, and finally a bond break to release the aldehyde. The ring-cleavage pathways identified here were consistent with the previously proposed cyclohexanecarboxylic acid degradation pathway [23, 30], in which multiple genes encoded the same reaction enzymes or isoenzymes. However, the enzyme specificity might be not particularly specific. In summary, multiple pathways and a variety of optional enzymes for degradation of NAs by strain CR3 guarantee its metabolic capacities in environments where a variety of hydrocarbons may serve as primary substrates for growth.

**Fig 7:**
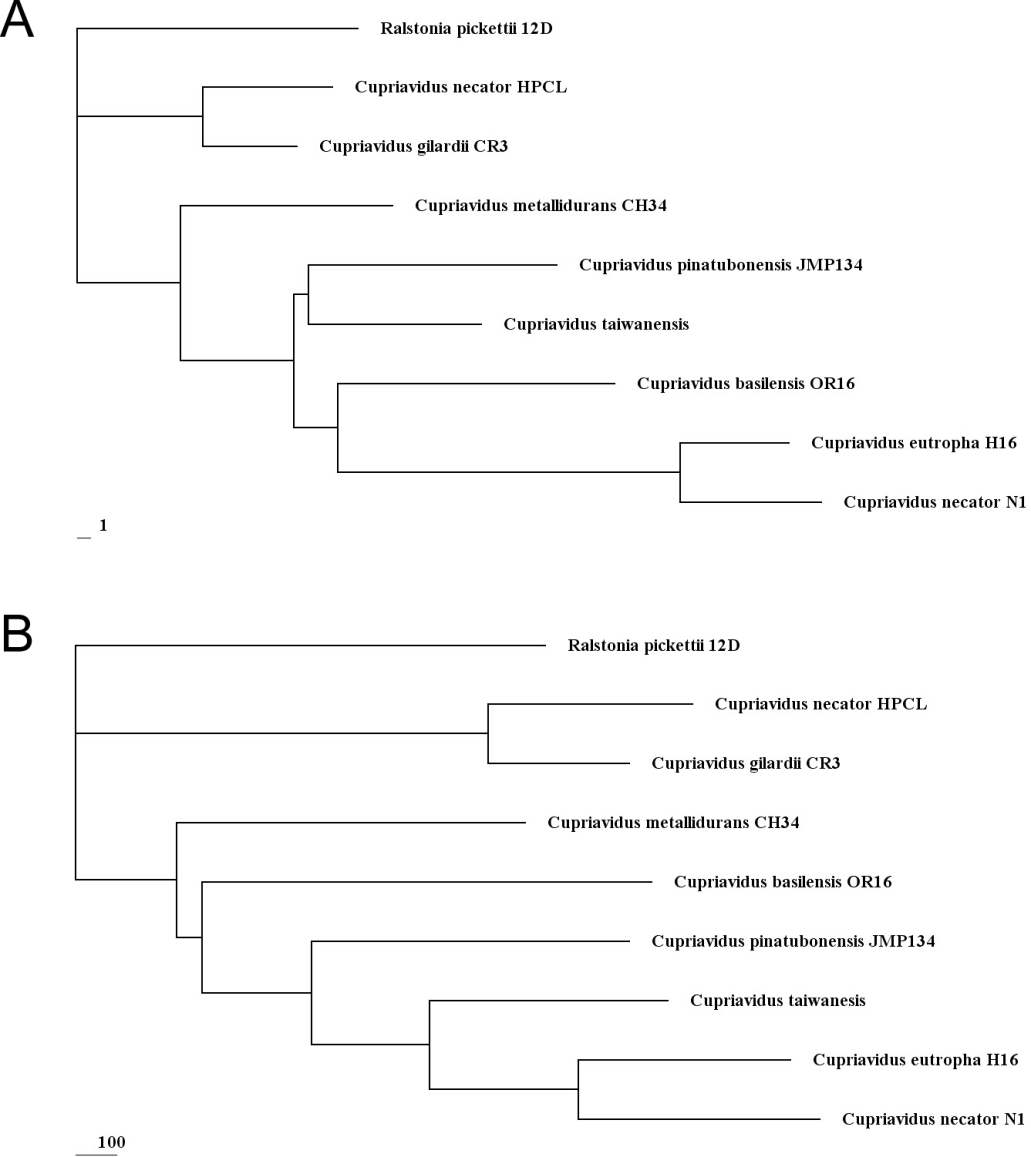
Rooted phylogenetic tree based on gene gain and loss. Cluster analysis was based upon the neighbour-joining method with *Ralstonia pickettii* 12D as the outlier. (A) Rooted phylogenetic tree based on NAs associated enzyme genes gain and loss; (B) Rooted phylogenetic tree based on genome genes gain and loss.

*Cupriavidus* species are able to degrade many different complex compounds [5–6, 15–18]. However, the degradation pathways have been unclear and the substrate is largely unknown. *C*. *gilardii* strain CR3 was able to use 4MCHCA and other similar molecules with branched alkyl groups (-(CH_2_)_m_CH_3_) as sole sources of carbon and energy for growth. This has never been found in any other strains of *Cupriavidus*. In an evolutionary tree based on gene gain and-loss, the branch length reflects the occurrence frequency of gene gain and-loss. The difference of occurrence frequency (gain and-loss) of genes related to NAs biodegradation (Fig 7A) was far more significant than that of genome-wide genes (Fig 7B). We suggest that NAs biodegradation capabilities in *Cupriavidus* have changed dramatically during evolution. The occurrence of gene gain and-loss in CR3 related to genes for enzymes participating in NAs biodegradation were less than in other *Cupriavidus* (Fig 7A). Moreover, enzyme-encoding genes in CR3 frequently occurred in gene clusters (two or more genes are adjacent) and involved 25.00% of these genes, while in other *Cupriavidus* strains, only 20.41% ~ 26.15% of enzyme encoding genes were clustered. Conserved enzyme genes in CR3 might increase the NAs degradation rate through a more compact gene organization to achieve genome structure optimization. Altogether strain CR3 is a good candidate for study of degradation pathways for model NAs or commercially available NA compounds.

### Metal resistance

*Cupriavidus* and other closely related bacteria possess a common characteristic of metal resistance [4–5, 8–9, 12–14]. Many genetic determinants support that CR3 strain would have potential resistance to heavy metals, including Cd^2+^, Zn^2+^, Co^2+^, Cu^2+^, Cu^+^, Hg^2+^, Pb^2+^, Ni^2+^, CrO_4_^2-^, Ag^+^, and possibly other heavy metals (Table 4). Some metal cations (e.g. Zn^2+^, Cu^2+^) are essential to living cells and function in enzyme-catalyzed reactions [52–53]. In contrast, other heavy metal cations as Ag^+^, Hg^2+^ are non-essential and considered toxic. Comparing to the model metal resistant strain CH34, strain CR3 carried many typical metal resistance loci such as *czcABC*, *copCBA* etc [4]. However, metal resistance mechanisms among diverse bacteria are slightly different for different metal tolerant strains. For instance, *pbrD* gene contained in CH34 [13] is absent in the CR3 *pbr* operon, while the other *pbrTRAB/C* genes are all present in CR3. *PbrD* gene is a Pb(II) binding protein involved in Pb(II). The absence of this gene would inhibit free export of Pb^2+^ and disable cyclical Pb^2+^ import and export, which would reduce accumulation of Pb^2+^ in CR3 cells [8]. In the case of resistance to mercury, the Hg^2+^ resistance system in CR3 consisted of the *merR* gene that regulates the operon, and the *merT* and *merP* genes that function to transport Hg^2+^ into the cell [8, 54]. The Ag^+^ resistance system was comprised of the central four *sil* genes, including *silA*, *silB*, *silR*, *silS*, while *silCEP* genes are absent. Here, strain CR3 presented two possible Ag^+^ resistance mechanisms: one involving an ATPase efflux pump, the other employing a chemiosmotic efflux pump [52, 55–56]. CR3 contained some classical ABC three-component resistance systems, most of them involving ATPase efflux pump systems, such as czcABC [10], cusCBA [57]. Other types of ion efflux systems were also identified in CR3 that export CrO_4_^2-^, Ni^2+^ etc. [4, 58–60]. Details on the major constitutive genes with putative roles in metal resistance are shown in Table 4. Based on current reports, *merP*, *pbrT*, and *chrF* genes are not required [61–63], but the *merT* gene is important in metal resistance [61, 64]. Comparing to other *Cupriavidus* strains, we find that *czc*, *pbr*, *chr*, *cus* genetic resistance determinants are common in all *Cupriavidus* bacteria. *Mer* is only present in CH34, OR16 and CR3, while the required *merT* gene was absent in the other five *Cupriavidus* bacteria. *ABC*.*PE* is present in CH34, H16, HPC (L), JMP134, N1 and CR3, while the required *ABC*.*PE*.*A* gene was absent in the *taiwanensis* and OR16 strains. *Cop* functions in CH34 and CR3, but it is not clear that whether the absent genes in the *cop* genetic determinant are essential. Likewise, mercury resistance is functional in the strains with no *silC* gene [65], which it is absent in *C*. *necator* HPC (L) strain, *C*. *pinatubonensis* JMP134 strain, and *C*. *taiwanensis* strain.

**Table 4:** Metal resistance gene operons in *C*. *gilardii* CR3.

Operon	Gene cluster	Gene identification number	Metal(s)
***czc***	*czcMNCBADRSE* ^*1*^; *czcCBA* ^*2*^; *czcI tolC czcBA* ^*2*^; *czcABC* ^*2*^	CR3_1349,1350,1352–1358; CR3_3402–3404; CR3_3627–3630; CR3_4522–4524	Cd^2+^, Zn^2+^, Co^2+^
***cop***	*copSRABCD* ^*1*^; *copQLFGJIDCBARS ompC copKBA* ^*1*^; *copKHFIDCBARS* ^*1*^	CR3_0172–0177; CR3_1309,1312–1315,1317–1323,1325–1328; CR3_1370–1372, 1374–1380	Cu^2+^, Cu^+^
***mer***	*merRTP* ^*1*^	CR3_2714–2716	Hg^2+^
***pbr***	*pbrAB/CRT* ^*1*^	CR3_1340–1342,1345	Pb^2+^
***sil***	*silABRS* ^*1*^	CR3_1305,1306,1322,1323	Ag^+^
***chr***	*chrFAB* ^*1*^	CR3_1391–1393	CrO_4_^2-^
***cus***	*cusFAB* ^*1*^	CR3_1304–1306	Cu^+^, Ag^+^
***ABC*.*PE***	*ABC*.*PE*.*A1AP1S* ^*1*^; *ABC*.*PE*. *A1AP1S* ^*1*^; *ABC*.*PE*.*SPP1A* ^*2*^; *ABC*.*PE*.*A1ASP1P* ^*2*^; *ABC*.*PE*.*AA1P1PS* ^*2*^	CR3_0010–0013; CR3_2425–2428; CR3_3225–3228; CR3_3295–3299; CR3_3708–3712	Ni^2+^

1. Gene operons were located at chr1 replicon.

2. Gene operons were located at chr2 replicon.

To validate metal resistance capacity in CR3, MIC tests of the predicted metals (Ag^+^, Cd^2+^, Co^2+^, Cr^6+^, Cu^2+^, Hg^2+^, Ni^2+^, Pb^2+^ and Zn^2+^) were performed. For comparison, the published MICs of the model metal resistant strain *C*. *metallidurans* CH34 [65] are also summarized at Table 5. *C*. *gilardii* CR3 showed resistance to all nine of the tested heavy metals. CR3 exhibited very high tolerance to Ag^+^, Cr^6+^, Hg^2+^, Pb^2+^ and Zn^2+^ compared with the model metal resistant strain *C*. *metallidurans* CH34. It is interesting to note that there is only one *Mer* gene cluster in CR3, while 4 *Mer* gene clusters in CH34. CR3 had the same MIC value for Cd^2+^ and Cu^2+^ as *C*. *metallidurans* CH34. Although there were 4 *czc* operons in CR3, the MIC values of Co^2+^ in CR3 were lower than those in CH34. It can be concluded that heavy metal resistance is a salient feature in CR3 and gene number is not proportional to the capacity of metal resistance.

**Table 5:** The minimum inhibitory concentration (MIC) of nine heavy metals in *C*.*gilardii* CR3 and C.*metallidurans* CH34.

		MIC(mM) ^a^	
Metal Ionic form	Formulation Compound	*C*. *gilardii* CR3	C. *metallidurans* CH34
**Ag** ^**+**^	AgNO_3_	0.06	0.0005
**Cd** ^**2+**^	CdCl25H_2_O	4	4
**Co** ^**2+**^	CoCl26H2O	2	25
**Cr** ^**6+**^	K2CrO4	2	0.4
**Cu** ^**2+**^	CuSO45H2O	3	3
**Hg** ^**2+**^	HgCl2	>0.04	0.0027
**Ni** ^**2+**^	NiCl26H2O	3	13
**Pb** ^**2+**^	Pb(NO_3_)_2_	4	1
**Zn** ^**2+**^	ZnSO47H2O	>24	12

^a^ MIC value of *C*.*gilardii* CR3 was from this study, MIC value of *C*. *metallidurans* CH34 was from reference [51].

The heavy metal tolerance of CR3 strain was mainly achieved by efficient ion efflux, metal-complexation and metal-reduction. The robust characteristics of this strain apparently result not only from its high-performance transport and detoxification systems for metal ions, but also from effective self repair mechanisms. The CR3 genome encoded direct repair protein genes, and genes that play roles in base-excision repair, mismatch repair (lack of *mutH* gene), nucleotide excision repair (a complete pathway). It also carried the complete RecFOR pathway to repair double strand DNA breaks by homologous recombination. The CR3 genome does not encode strand discrimination factor *mutH* gene as in the CH34 genome [4], nor a known DNA adenine methylase and DNA patch repair protein. The supplementary genes of other mismatch excision repair factors, *ssb* (CR3_0318) and *uvrD* (CR3_0231, CR3_1976), might complement mismatch repair function in CR3. Both direct resistance mechanisms and self repair mechanisms safeguard the ability of strain CR3 to tolerate the harsh environment presented by life in natural asphalt deposits.

## Supporting Information

S1 Fig Synteny plot analysis of Cupriavidus chromosome 2. (TIF)

S2 Fig Gene function annotation of CR3 genes based on GO annotation. **(A). GO function distribution of CR3 genes based on level 1 classification; (B). Significantly enriched GO function of CR3 unique genes based on level 3 classification.** The significance is characterized by *p_*value, based on chi-square test.
(TIF)

S3 Fig KEGG pathway and pathway enrichment analysis of CR3 genes. **(A). KEGG pathways that CR3 genes mainly participate in; (B). KEGG pathway enrichment analysis of CR3 unique genes.** The significance is characterized by *p* value, based on chi-square test.
(TIF)

S4 Fig Functional distribution of CR3 genes based on COG classification. **(A). Primary COG classification; (B). Significant secondary COG features.** The significance is characterized by *p*_value, based on chi-square test.
(TIF)
