# Supplementary figures and images for: Genome Sequence Analysis of the Naphthenic Acid Degrading and Metal Resistant Bacterium Cupriavidus gilardii CR3
Source: PLoS One. 2015 Aug 24;10(8):e0132881. doi: 10.1371/journal.pone.0132881 (PMC4547698; doi:10.1371/journal.pone.0132881)

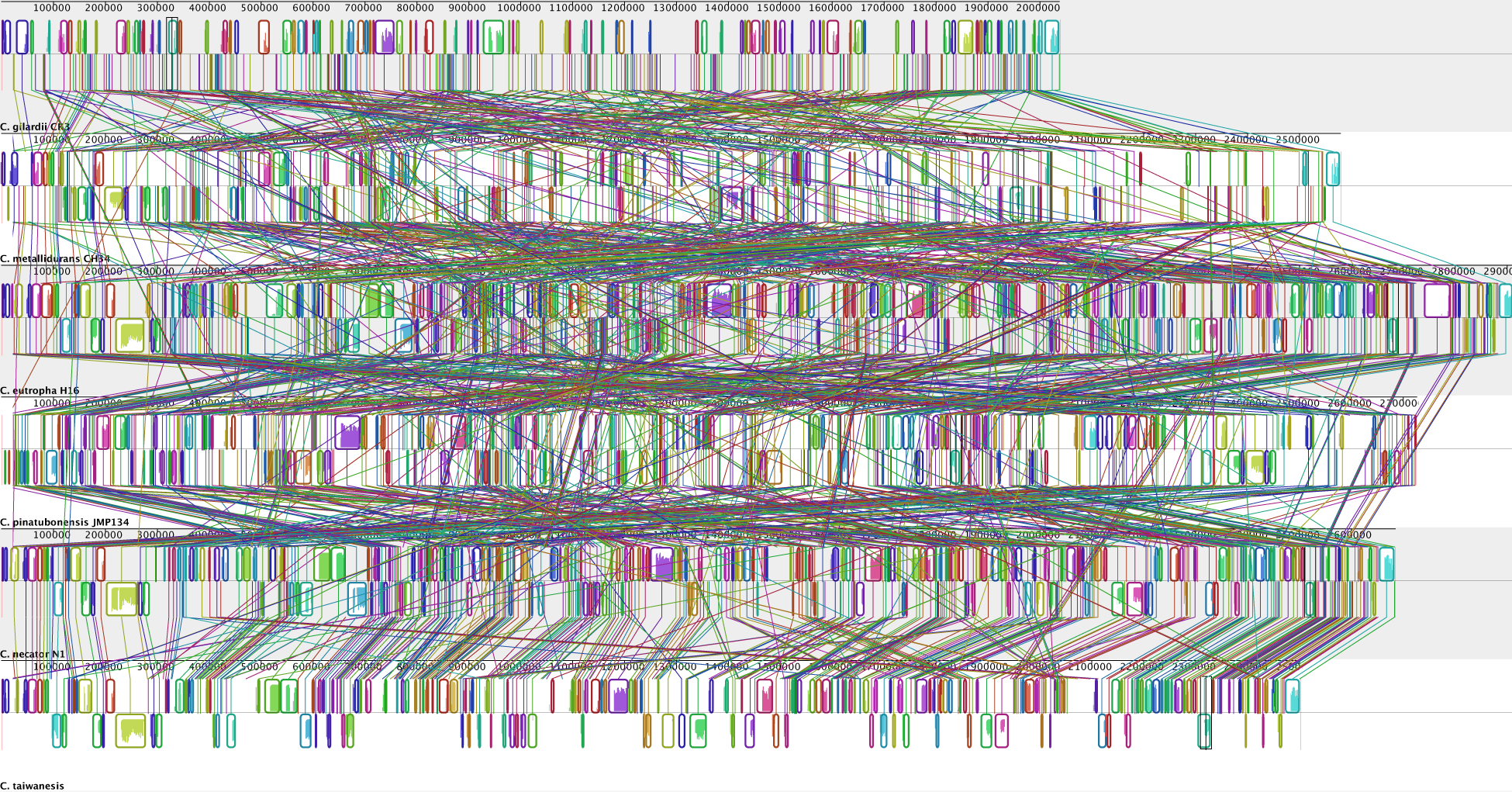

Supplement: S1 Fig — (TIF) [file pone.0132881.s001.tif]

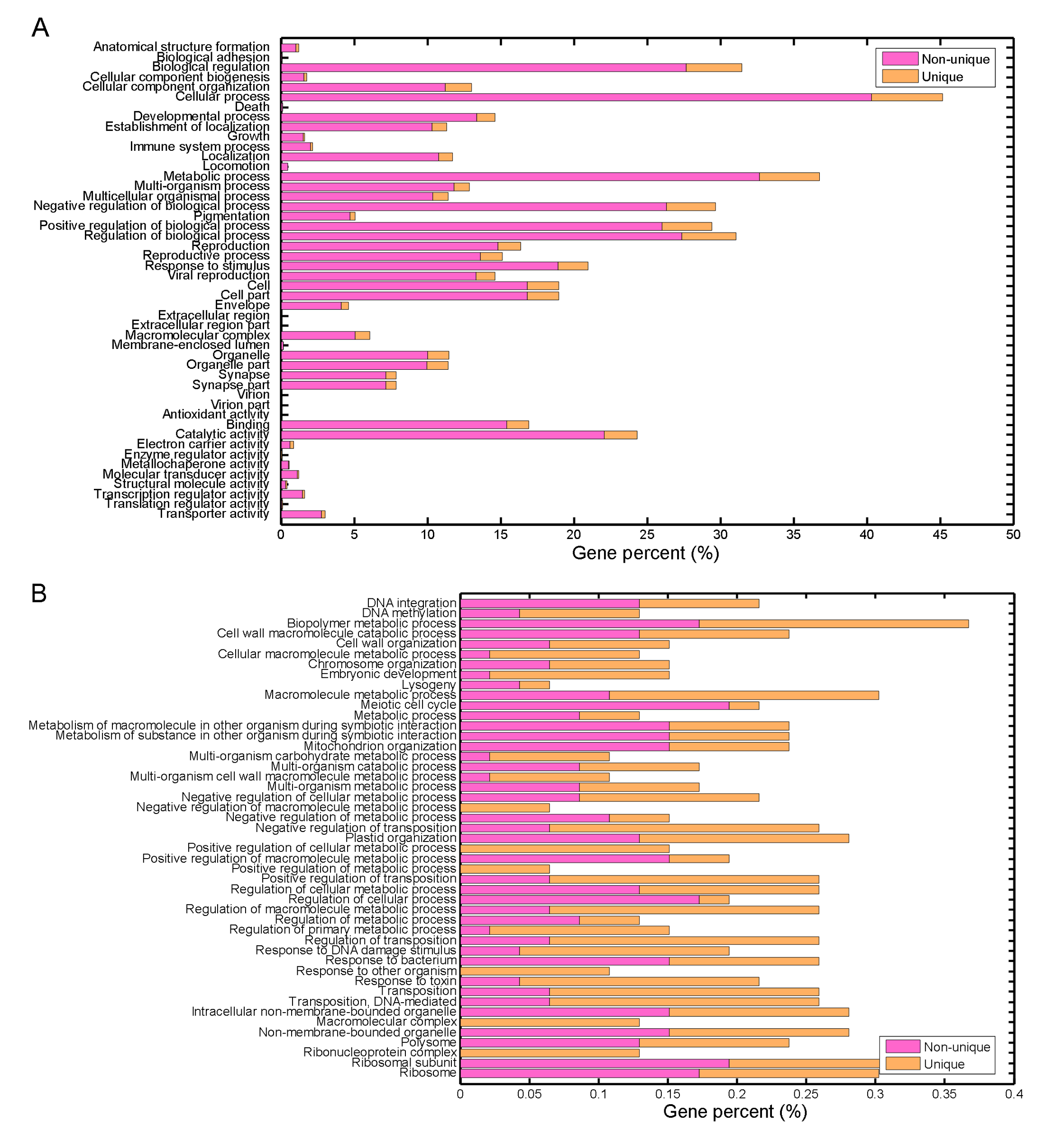

Supplement: S2 Fig — (A). GO function distribution of CR3 genes based on level 1 classification; (B). Significantly enriched GO function of CR3 unique genes based on level 3 classification. The significance is characterized by p_value, based on chi-square test. (TIF) [file pone.0132881.s002.tif]

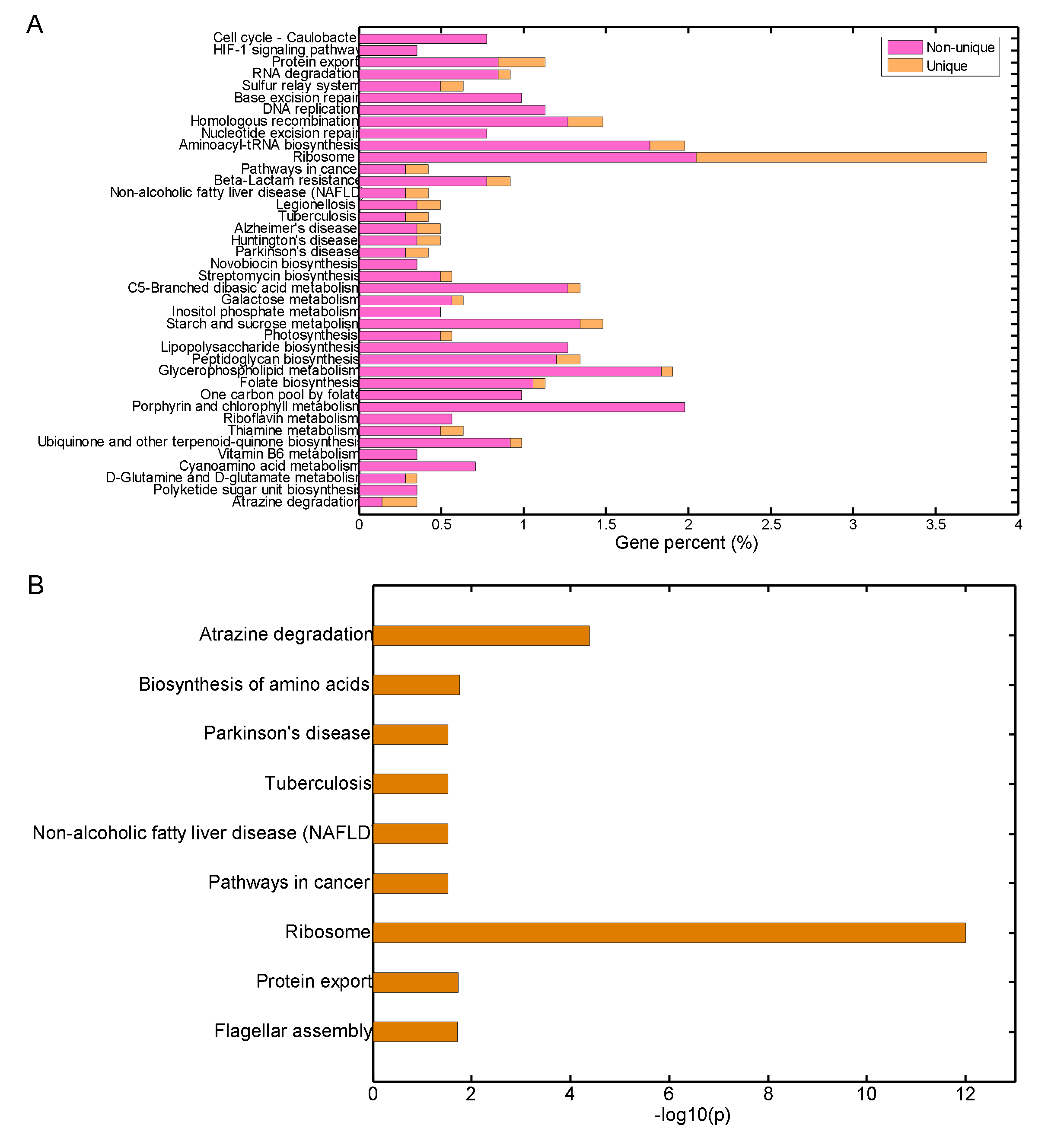

Supplement: S3 Fig — (A). KEGG pathways that CR3 genes mainly participate in; (B). KEGG pathway enrichment analysis of CR3 unique genes. The significance is characterized by p value, based on chi-square test. (TIF) [file pone.0132881.s003.tif]

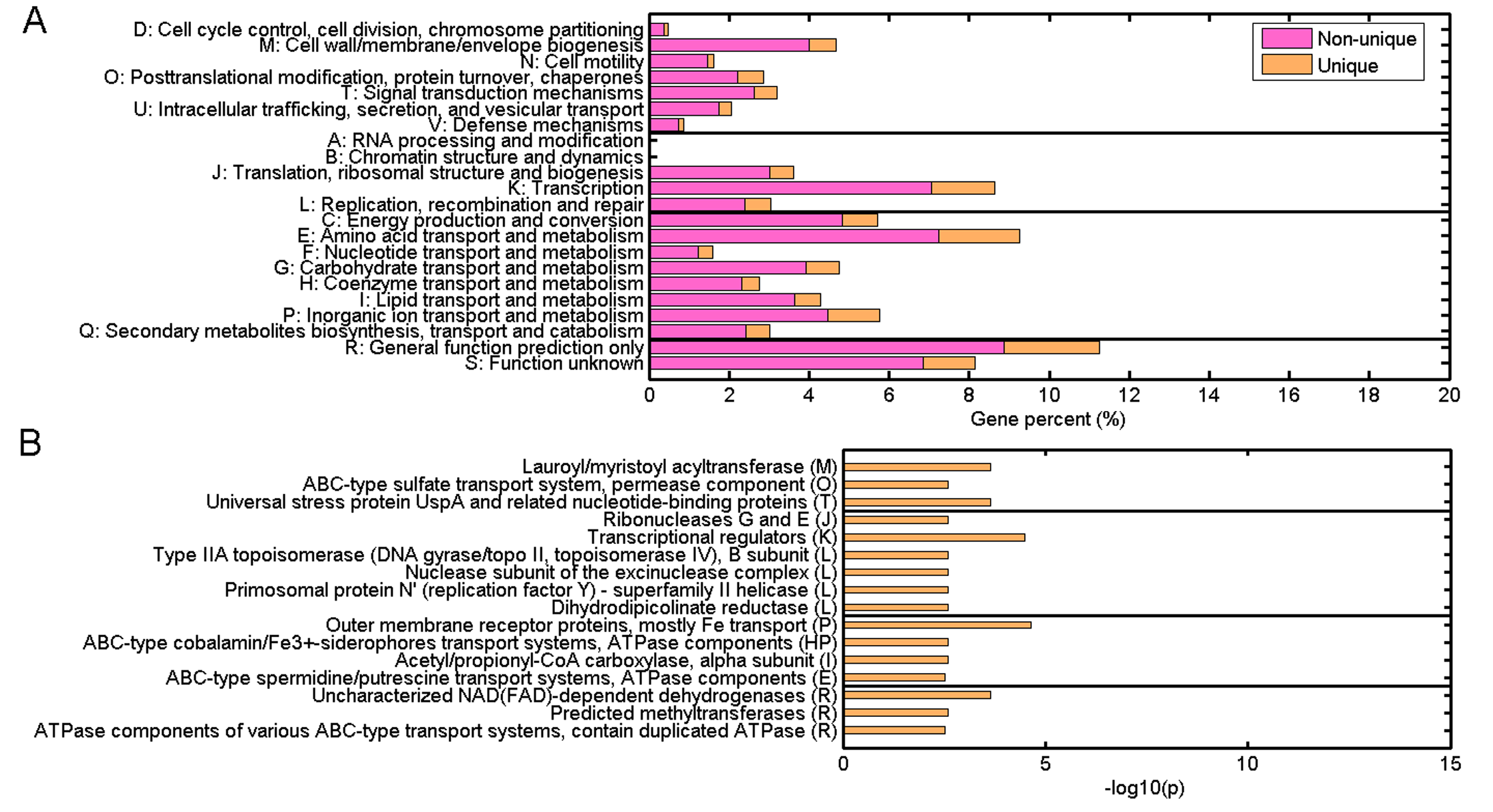

Supplement: S4 Fig — (A). Primary COG classification; (B). Significant secondary COG features. The significance is characterized by p_value, based on chi-square test. (TIF) [file pone.0132881.s004.tif]
